# Utilizing Experimental Mouse Model to Identify Effectors of Hepatocellular Carcinoma Induced by HBx Antigen

**DOI:** 10.3390/cancers12020409

**Published:** 2020-02-10

**Authors:** Ming-Hui Yang, Marcelo Chen, Hsiao-Hsuan Mo, Wan-Chi Tsai, Yu-Chi Chang, Chin-Chuan Chang, Ko-Chin Chen, Hsin-Yi Wu, Cheng-Hui Yuan, Che-Hsin Lee, Yi-Ming Arthur Chen, Yu-Chang Tyan

**Affiliations:** 1 National Mosquito-Borne Diseases Control Research Center, National Health Research Institutes, Miaoli 350, Taiwan; w3e3@hotmail.com; 2 Master Program in Clinical Pharmacogenomics and Pharmacoproteomics, College of Pharmacy, Taipei Medical University, Taipei 110, Taiwan; 3 Department of Medical Education and Research, Kaohsiung Veterans General Hospital, Kaohsiung 813, Taiwan; 4 Department of Urology, Mackay Memorial Hospital, Taipei 104, Taiwan; mchen4270@yahoo.com; 5 Department of Cosmetic Applications and Management, Mackay Junior College of Medicine, Nursing and Management, Taipei 112, Taiwan; 6 School of Medicine, Mackay Medical College, New Taipei City 252, Taiwan; 7 Department of Medical Imaging and Radiological Sciences, Kaohsiung Medical University, Kaohsiung 807, Taiwan; mo840706@gmail.com; 8 Department of Medical Laboratory Science and Biotechnology, Kaohsiung Medical University, Kaohsiung 807, Taiwan; wanchi@kmu.edu.tw; 9 Department of Medicine, Kaohsiung Medical University, Kaohsiung 807, Taiwan; cs342070@gmail.com (Y.-C.C.); chinuan@gmail.com (C.-C.C.); 10 Department of Nuclear Medicine, Kaohsiung Medical University Hospital, Kaohsiung 807, Taiwan; 11 Department of Pathology, Changhua Christian Hospital, Changhua 500, Taiwan; 148350@cch.org.tw; 12 Instrumentation Center, National Taiwan University, Taipei 106, Taiwan; hsinyiwu@ntu.edu.tw; 13 Mass Spectrometry Laboratory, Department of Chemistry, National University of Singapore, Singapore 119077, Singapore; chmyuch@nus.edu.sg; 14 Department of Biological Science, National Sun Yat-sen University, Kaohsiung 804, Taiwan; chlee@mail.nsysu.edu.tw; 15 Department of Medical Research and Education, Cheng Hsin General Hospital, Taipei 112, Taiwan; 16 Graduate Institute of Medicine, College of Medicine, Kaohsiung Medical University, Kaohsiung 807, Taiwan; 17 Institute of Medical Science and Technology, National Sun Yat-sen University, Kaohsiung 804, Taiwan; 18 Department of Medical Research, Kaohsiung Medical University Hospital, Kaohsiung 807, Taiwan; 19 Center for Cancer Research, Kaohsiung Medical University, Kaohsiung 807, Taiwan; 20 Research Center for Environmental Medicine, Kaohsiung Medical University, Kaohsiung 807, Taiwan

**Keywords:** HBx, missing proteins, hepatocellular carcinoma

## Abstract

Hepatocellular carcinoma (HCC) is among the ten most commonly diagnosed cancers and the fourth leading cause of cancer-related death. Patients with hepatitis B virus (HBV) infection are prone to developing chronic liver diseases (i.e., fibrosis and cirrhosis), and the HBV X antigen plays an important role in the development of HCC. The difficulty in detecting HCC at the early stages is one of the main reasons that the death rate approximates the incidence rate. The regulators controlling the downstream liver protein expression from HBV infection are unclear. Mass spectrometric techniques and customized programs were used to identify differentially expressed proteins which may be involved in the development of liver fibrosis and HCC progression in hepatitis B virus X protein transgenic mice (HBx mice). FSTL1, CTSB, and TGF-β enhanced the signaling pathway proteins during the pathogenesis of HBx. Missing proteins can be essential in cell growth, differentiation, apoptosis, migration, metastasis or angiogenesis. We found that LHX2, BMP-5 and GDF11 had complex interactions with other missing proteins and BMP-5 had both tumor suppressing and tumorigenic roles. BMP-5 may be involved in fibrosis and tumorigenic processes in the liver. These results provide us an understanding of the mechanism of HBx-induced disorders, and may serve as molecular targets for liver treatment.

## 1. Introduction

Globally, hepatocellular carcinoma (HCC) is among the top ten most commonly diagnosed cancers and the fourth leading cause of cancer-related death. In males, the mortality rate of HCC has been increasing and HCC is ranked as the second most common cause of cancer death [1]. Patients with hepatitis B virus (HBV) or hepatitis C virus (HCV) infection are at high risk of HCC development. An HBV infection can be acute or chronic. It has been reported that patients chronically infected with HBV may bear a 5- to 100-fold increased risk of HCC development [2]. In order to control HBV, an orally bioavailable antiviral prodrug of tenofovir named Tenofovir alafenamide (TAF, a nucleotide reverse transcriptase inhibitor) has been characterized with improved renal and bone safety in clinical trials [3]. Within the content of HBV, one of the cutting edge developments in HCC prevention is to use the Toronto HCC Risk Index (THRI) to assess the disease-specific incidence of HCC from cirrhosis patients for HCC risk prediction. The THRI combines clinical and laboratory parameters and has been validated by another cohort for its good predictive ability for HCC [4]. Additionally, HBV-related tumors have been characterized with high rates of chromosomal alterations and p53 inactivation, overactivation of fetal liver progenitor cells, and AKT pathway and β-catenin mutations [5].

HBV is a DNA virus and its genome can be converted into covalently closed circular DNA (cccDNA) and it can serve as a transcriptional template [6]. cccDNA integrates its DNA into the host genome resulting in both genomic instability and mutagenesis of diverse cancer-related genes. cccDNA constantly expresses the HBV regulatory HBx protein (Hepatitis B virus X antigen, a protein of 154 amino acids) and unleashes the cellular transcription program and proliferation control via oncogenic Ras signaling [7]. As a result, hepatocytes are sensitized to carcinogenic factors. HBx also induces anti-apoptotic activity and promotes the cell cycle progression of hepatocytes through the Akt/mTOR pathway [8]. HBx has been proposed as a multifunctional protein because HBx is required for virus replication and its effects on apoptosis and metabolism are likely to be essential for management of chronic HBV infection [9].

HBV infection is heavily involved in human hepatocarcinogenesis, and the Hepatitis B virus X antigen plays the lead role [10]. It has been reported that transgenic mice using the albumin promoter to express HBx were shown to develop HCC at 14 to 18 months without additional treatment [11,12]. Although there were no cancer cells detected in the liver of these HBx transgenic mice at the 11–13 months stage, liver cancer cells were detected later on. This is an ideal timing to study proteins involved in the transition from non-tumor to tumor and to investigate the critical changes of HBx-induced HCC carcinogenesis.

Biomarkers are biochemical responses which can be indicators of exposure to and/or effects of a stressor within an individual. Missing proteins altered in concentration or state according to a specific biological process or disease fit into this category since measurement of concentration changes, relative or absolute, is essential to the discovery of valid biomarkers [13]. Biomarkers found in vitro or in vivo require further validation, and biobanking for viral hepatitis research has been well established [14]. This will facilitate the translational process. There are several possible reasons for missing proteins to go undetected (without evidence at the protein level); these can be technical (such as ionization/detection failure or lack of uniquely identifiable tryptic peptides), biological (low abundance in sample, silent genes present only in the genome, expressed only developmentally regulated or only in a certain time or space) or informatics (erroneous annotation, parsimonious protein assembly of MS/MS identifications or expression in rare cell/tissue types) [15].

Missing proteins may be involved in various cellular activities in both normal and tumor cells. They may function in cell growth, division, differentiation, apoptosis, migration, metastasis, angiogenesis and adhesion [16]. Thus, missing proteins in tumor-developing tissue could be potential biomarkers associated with carcinogenesis. Additionally, disease-specific protein biomarkers could help us understand disease mechanisms in which proteins play a key role. Previously, we generated a database listing the identities of missing proteins in tumor cells and potential diagnostic cancer biomarkers [17]. In addition, an HBx-induced HCC mouse model has been demonstrated to identify potential chemopreventive agents of HBV-related hepatocarcinogenesis [18]. In this study, we used proteomic approaches to characterize proteins significant for the development of liver fibrosis and transition to HCC. The expression profile offers not only information on the nature of missing proteins related to liver physiology, but also potential diagnostic protein markers involved in the progression of HBx-induced HCC. These markers might serve as potential therapeutic target molecules for HCC treatment. 

## 2. Results 

### 2.1. Pathological Findings in HBx Mice

**Figure 1:**
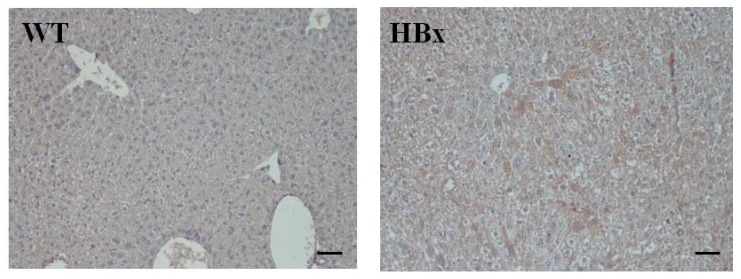
Immunohistochemistry (IHC) of mouse liver samples from wide type (WT) and hepatitis B virus X protein transgenic (HBx) mice. Samples were stained with antibodies of HBx. The scale bars indicate 30 μm.

Figure 1 shows the immunohistochemistry staining of mouse liver samples from WT and HBx groups. Samples were stained with antibodies of HBx. Expression of HBx protein in the transgenic livers was confirmed by immunohistochemistry (IHC) using an HBx polyclonal antibody. Compared with liver tissue of WT littermate mice, a higher proportion of HBx protein-expressing levels was observed in HBx mice. Enzymes produced from the liver, such as ALT and AST, can profile liver function. Figure S1 shows the AST and ALT averages in wild-type and HBx mice. The AST and ALT averages were higher in HBx mice than in wild-type mice. AST is found in several organs, such as the liver, muscle and heart, whereas ALT is mainly found in the liver and kidneys. Also, ALT is mainly distributed in the cytoplasm of hepatocytes, while AST is mainly distributed in the cytosol and mitochondria of hepatocytes. Thus, the index of ALT in liver injury is more sensitive and specific than that of AST, and it is often used as one of the indicators during HBV assessment. 

**Figure 2:**
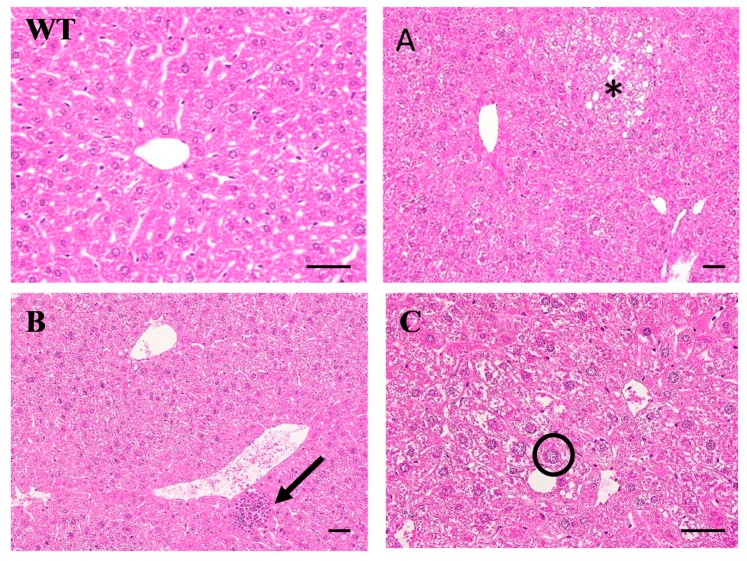
Hematoxylin and eosin staining histopathological examination of the livers from WT and HBx mice. (**A**) Focus of hepatocellular alteration (Clear cell foci); (**B**) inflammatory cell infiltration; (**C**) hepatocellular hypertrophy. The scale bars indicate 30 μm.

**Figure 3:**
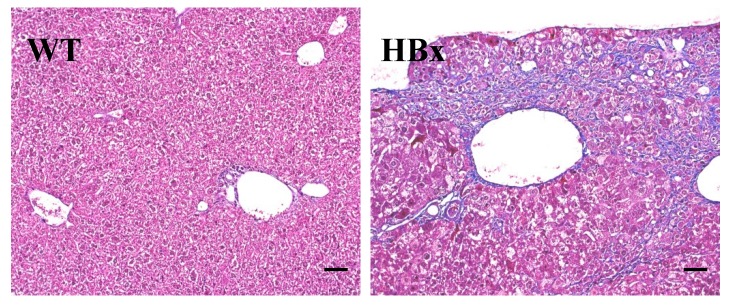
Masson’s trichrome staining for the liver histopathological examination of the livers from WT and HBx mice. (HBx) Diffuse fibrosis of hepatic subcapsule and fibrosis expansion without septa formation.

All the liver tissues were processed for Hematoxylin and Eosin (H&E) staining for histological analysis while only representative results are shown in Figure 2. There are some microscopic lesions in the livers of mice, including (1) changes in hepatocyte focus (clear cell lesions), as shown in Figure 2A, (as indicated by*); (2) inflammatory cell infiltration in the liver, as shown in Figure 2B (as indicated by bold arrows); (3) hepatocyte hypertrophy (as indicated by arrows) and nuclear giant cell hepatocytes (as indicated by a circle), as shown in Figure 2C; and (4) hepatic ellipsoidal hyperplasia. Other observations include the accumulation of glycogen in the liver, cell swelling, granular or cell-like appearance of the cytoplasm, and vacuoles lacking uniform size. Figure 3 shows the Masson’s trichrome staining for the liver histopathological examination of the livers from WT and HBx mice. The HBx liver sample showed diffuse fibrosis of hepatic subcapsule and fibrosis expansion without septa formation. 

### 2.2. Proteomic Analysis

**Figure 4:**
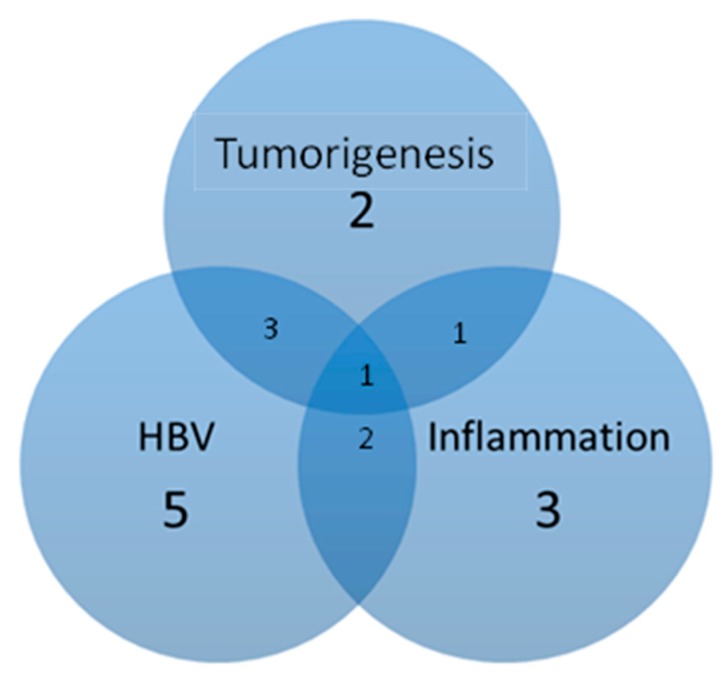
The MASCOT results indicate that 11 proteins were associated with HBV, 7 proteins were associated with inflammation, and 7 proteins were associated with tumorigenesis. Among these proteins, Cathepsin B (CTSB) and Follistatin-related protein 1 (FSTL1) were involved in tumorgenesis, but not HBV nor inflammation. Transforming growth factor-beta receptor-associated protein 1 (TGF-β) was the only protein associated with tumor, inflammation, and HBV.

Thousands of unique peptides were identified in pooled liver samples from the MASCOT database. These proteins were identified at minimal confidence levels as only one unique peptide sequence was matched. Studies reported a total of 541 protein identifications with high confidence levels. In this study, HBx mice were selected to profile HBV-related proteins. Hepatic proteins from both HBx and wild-type mice were extracted, digested and applied to a nano-UPLC-ESI-MS/MS system for analyzing fragmentation patterns of tryptic peptides. Each sample was subjected to three replicate runs. A total of 16 proteins were related to HBV-infection, tumorgenesis and inflammation. The search results of the liver samples from HBx group showed that Cathepsin B (CTSB) and Follistatin-related protein 1 (FSTL1) were the only proteins involved in tumorigenesis (Table 1 and Table 2), while Cytochrome c (CYC), Dihydrolipoyl dehydrogenase (DLDH), Exocyst complex component 4 (EXOC4), Glutamate–cysteine ligase catalytic subunit (GSH1) and Ubiquitin-40S ribosomal protein S27a (RS27A) were involved in HBV-infection (Table 3 and Table 4). heat shock 70 kDa protein 1-like (HS71L), heat shock protein HSP 90-beta (HS90B) and heat shock cognate 71 kDa protein (HSP7C) were involved in inflammation (Table 5 and Table 6). Among these proteins, only transforming growth factor-beta receptor-associated protein 1 (TGF-β) was associated with tumorigenesis, inflammation, and HBV-infection (Figure 4). 

**Figure 5:**
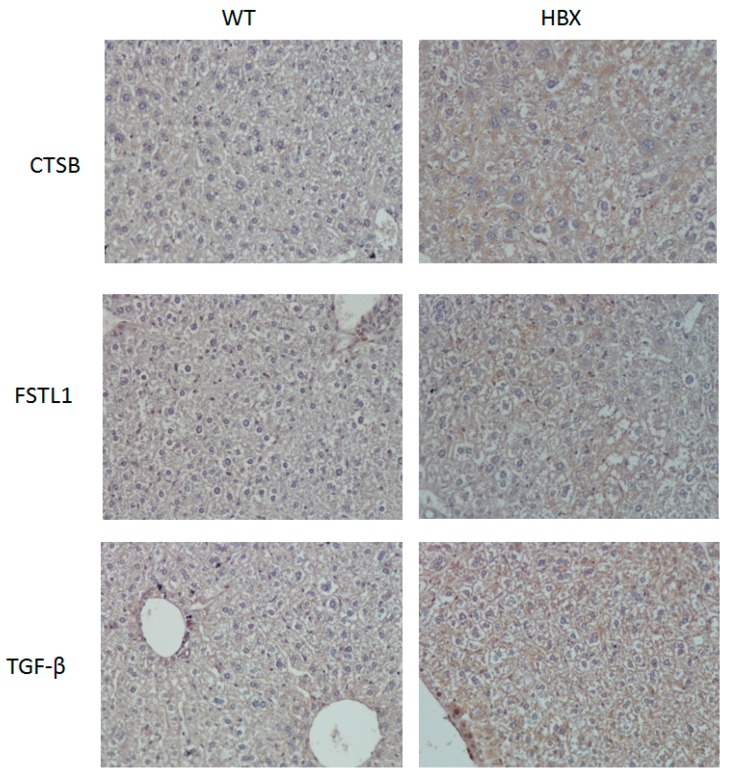
Immunohistochemistry (IHC) of mouse liver samples from WT and HBx groups. Samples were stained with antibodies of Cathepsin B (CTSB), Follistatin-related protein 1 (FSTL1) or transforming growth factor-beta receptor-associated protein 1 (TGF-β.)

**Figure 6:**
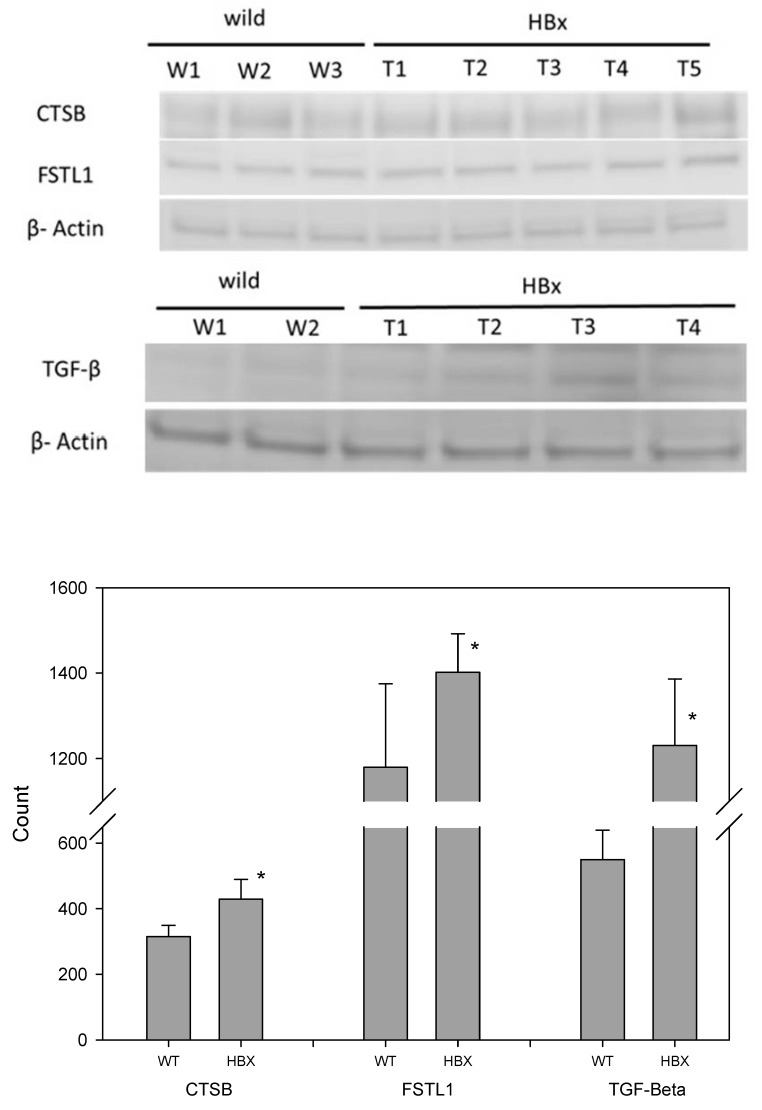
Western blotting of mouse liver samples from WT and HBx groups. Samples were stained with antibodies of CTSB, FSTL1, TGF-β and β-actin (N = 8, * *p* < 0.05).

The expressions of three tumor-associated proteins, TGF-β, CTSB and FSTL1, were examined using immunohistochemical staining and Western blotting (Figure 5 and Figure 6). Samples from HBx mice were more hypertrophic and had larger nuclei than those from WT mice; the antibodies of these three proteins were also evident in the liver cells of these HBx mice.

The differential protein expressions of CSTB, FSTL1 and TGFb were detected by three methods. First, they were distinguished by mass spectrometry as a qualitative analysis. Liver lysates of HBx mice and those of WT mice were pooled to reduce individual differences. Then, an immunohistochemical (IHC) technique was applied. Each liver was sliced into several sections and the differential protein expression between HBx and WT groups were easily visualized. Western blotting was also performed. Liver lysates of HBx mice and those of WT mice were also pooled. Since Western blot is a semi-quantitative analysis and the abnormal hepatic area was much smaller than the normal area, the differentially expressed protein signals may have been diluted. Moreover, the appearance of the liver was not significantly different between the two groups, and not all livers of HBX mice were diseased uniformly. IHC photos represent tissue microenvironments. The images taken were those of abnormal liver tissues, but that does not mean that the entire liver was abnormal. IHC is very different from Western blot, as Western blot is based on tiny pieces of liver grinded for proteins. Because some of the abnormal liver tissues were only pre-cancerous, they were hard to distinguish from normal liver tissue grossly. The livers of HBX mice were mostly normal. Proteins that appeared to be uniform under the microscope were only partly mottled in the entire liver. Normal tissue may have been sampled, so the specific protein differences may have been diluted in Western blot. The images of Western blots are not easy to identify the density with the naked eyes, but the image software analysis can still identify the difference in density of each band. In this study, Western blots of eight pairs of liver tissues of HBX and WT mice were compared, and were repeated three times for each liver sample. The concentrations of CSTB, FSTL1 and TGF-β were significantly different (*p* < 0.05). Mass spectrometry and immunohistochemistry showed that the protein expressions of CSTB, FSTL1 and TGFb were notably different between WT mice and HBx mice.

Previously, our group reported that the expression of hepatic glycine n-methyltransferase (GNMT) was down-regulated in NAFLD and HCC [19,20]. We also found that an oncoprotein–Phosphatidylinositol-3,4,5-Trisphosphate Dependent Rac Exchange Factor 2 (PREX2) was a GNMT-interacting protein. The degradation of PREX2 was much slower in the absence of GNMT, and this could lead to HCC development. Assessment of the hepatic PREX2 expression in HBx mice showed that the expression of PREX2 in HBx transgenic mice was higher than that in WT mice. (Figure S2).

### 2.3. Missing Protein Analysis

In this study, the peptides identified from MS/MS spectra were applied to our search program made by JAVA programming with the gene expression sequences downloaded from the HPA database. This database provides rapid analysis of suspected missing and non-missing proteins from chromosomes. A missing protein was positively identified when two or more product ion mass spectra of peptides completely matched the sequence of a missing protein in the database.

After utilizing the program, there were 29 possible missing proteins in the HBx group (Table 7). A further investigation found that 19 of them were missing proteins at the PE2 level. Among those 29 proteins, 13 proteins were related to tumor study, in which eight proteins were tumorigenic. CCDC67-002, GDF11-001, and LMX1A-001 were identified at the protein level and BMP5, FEZF2-004, HS6ST3, KIAA2022-001 and SP5 were missing proteins. Three were tumor suppressors (ADAMTS15, OSR1 and USP27X) and one was down-regulated in HCC (SRCIN1-001). In addition, LHX2-004 was related to HCC fibrosis (Table 8).

## 3. Discussion

Liver enzymes, such as ALT and AST, can sketch the liver function. Our data showed the AST and ALT averages were higher than in wild-type mice. For example, mild ALT elevation (5 times higher than normal) was reported in unspecific liver diseases, and moderate ALT elevation (normally 5 to 20 times) was common in acute viral hepatitis or drug-induced hepatitis, and severe ALT elevation (normally 20 to 50 times) was mainly seen in acute viral hepatitis. However, it has been stated that AST is also a good predictor in evaluating the performance of AST in low-infectious hepatitis B [21]. Since damaged livers secrete more AST and ALT, the abnormally high levels of AST and ALT indicate liver damage. The AST level was elevated in 34% of HBx mice. In addition, the ALT level was elevated in 90% of HBx mice. These were suggestive of either liver fibrosis or chronic hepatitis. 

Our H&E staining of the liver tissues from HBx mice showed some microscopic lesions. These were consistent with the results mentioned in the literature [22]. These HBx mice have liver damage with cell morphology changes, but no liver tumors. Thus, these HBx mice were still in the stage where HBx virus was still replicating and none of the liver tumors had been formed.

Hepatocellular carcinoma has been described by Debes and coworkers as a unique tumor lacking biomarkers due to multifaceted complexities. This includes highly variable genetic components and variable liver disease-dependent protein expression as well as the unique individual immune system since an inflammatory environment leads to cirrhosis before forming HCC [23]. In this study, hepatic proteins from HBx mice have been characterized for biomarkers. Cathepsin B is a lysosomal cysteine protease and is one of 11 cysteine cathepsins [24]. Cathepsin B can function both as an endopeptidase and an exopeptidase (with carboxydipeptidase activity) [25]. Elevated expression of Cathepsin B protein was found in studies of breast, thyroid, and colorectal cancers [26,27,28]. Such high expression of cathepsin B in many different cancers led to the assumption that this enzyme played a fundamental role in progression of these tumors. Indeed, experimental down-regulation of Cathepsin B demonstrated reduced motility and invasion of cancer cells [29]. However, the role of Cathepsin B in HCC is still unknown. Ruan and colleagues reported that the over-expression of cathepsin B in HCC was associated with poor prognosis while Qin and coworkers stated that HCC patients with low expression levels of CTSB had poor survival [30,31].

Follistatin-like 1 (FSTL1) has calcium ion and heparin binding functions and five domains including a FS-like and a Kazal-like domain, two EF-hand domains, and a von Willebrand factor type C domain [32]. It belongs to the secreted protein acid and rich in cysteine (SPARC) family and it exhibits variable expression during pathological conditions including cancer. Multiple signaling pathways and biological processes are involved in FSTL1-mediated vascularization and regulation of immune responses. However, results from cancer progression studies of the proliferative, apoptotic, migratory, and inflammatory effects of FSTL1 were inconsistent [33]. Increased expression of FSTL1 was observed in most cases of HCC when compared to healthy controls. Overexpression of FSTL1 resulted in a HCC cell line (Huh7) expansion, seen as increased proliferation and inhibited apoptosis. AKT/GSK-3β signaling was activated, and the expression of pro-apoptotic BIM and BAX proteins decreased [34].

The TGF-beta (β) family is not only multifunctional, but also essential for survival. It plays important roles in regulation of growth, development of tissues, and maintenance of homeostasis [35]. This family has three members –TGF-β1, β2 and β3 and TGF-β 1 is the most abundant and universally expressed member. TGF-β 1, a secreted cytokine inactive during storage in the extracellular matrix, regulates growth and differentiation in a variety of cell types and is involved in normal development and immune function. However, it also triggers the epithelial-mesenchymal transition, angiogenesis, invasion, and connective tissue growth factor production [36,37,38,39]. These features promote HCC tumor progression. Fibrosis, epithelial-mesenchymal-transition and TGF-beta are closely related to cancer progression, and cancer progression is mediated by cancer-associated fibroblasts (CAF) rendering drug resistance. The tumor microenvironment may play a key role in drug resistance and the central element of the tumor microenvironment is a cancer-associated fibroblast (CAF). The mechanisms of CAF-mediated therapeutic resistance include HGF/MET by restoring survival signaling pathway (PI3K or MAPK), PDGF/PDGF-R by improving IFP, IL-6 by activating of STAT3, SDF-1/CXCR4 by BCL-2 / BCL-XL or activating NOTCH pathway to regulate CSCs and AnXA1/TGR-beta/CA IX/ MMP-2/MMP-9 to regulate EMT [40]. Indeed, it has been shown that CAFs and TGF-β signaling activation contribute to chemotherapy resistance in cancer [41]. Another study showed that HCC patients receiving sorafenib may benefit from immune modulation strategies [42]. Argentiero and colleagues reported that a specific WNT pathway inhibitor may instruct the immune system to increase cytotoxicity in tumor and re-establish the anti-PDAC immunity [43]. Such immune-based therapeutic strategies targeting WNT may be adapted to hepatic fibrosis. However, HCC can be classified by immune status, where roughly 30% of HCCs belong to the "immune class” (inflamed hot tumors) and the rest are “non-inflamed” or “cold tumors” [44]. These hot HCC tumors are more likely to be effective if treated with immune checkpoint inhibitors. Half of the cold tumors belong to the "immune exclusion class", which is characterized by Wnt/CTNNB1 mutations and might have innate resistance to anti–PD-1/PD-L1 inhibitors. The rest, about one third of HCCs, are categorized as "immune intermediate class" (noninflamed tumors with wild-type CTNNB1 and intermediate intensities of immune infiltration). Since the molecular pathways and gene signatures are not defined, the patients may or may not respond to immunotherapy. Thus, selections of patients and trials are definitely very important for HCC treatments because intra-individual differences may influence the outcome. For example, the importance of adequate patient selection in sorafenib treatment for HCC has been shown in a recent clinical trial where sorafenib usage was restricted to Child–Pugh A patients [45].

TGF-β is also heavily involved in tumor microenvironment. There is increasing attention to the role of the tumor microenvironment in cancer development and progression. Because cancer cells build up a complex relationship within the surrounding environment and the host immune response is important to the success of immunotherapy, the tumor vasculature is the key to successful treatment response. Tumor microenvironment may be affected by the types of tumors. In liquid type cancers, endothelial cells play an important role. Endothelial cells regulate the microenvironment through immune cell trafficking and immune response modulation. Such response can be a powerful treatment approach [46]. In fact, endothelial cells are important components of the bone marrow microvasculature and are in close contact with CD8+ T cells. Moreover, endothelial cells trap and present antigen to CD8+ T cells as well as produce high amounts of IL-10 and TGF-β. However, multiple myeloma patient bone marrows produce tumor-specific CD8+ T cells that are unable to control the proliferation of the malignant plasma cells. Furthermore, there are two CD8+ T cell populations in multiple myeloma patient’s bone marrow. One is sustained by endothelial cells and produce TGF-β to promote the development of tumors and to counteract the activity of the other population. It illustrates an unpredicted immune regulatory mechanism that inhibits the development of antitumor immunity and may diminish the effect of immunotherapy [47]. In agreement with the idea of microenvironment, targeting the TGF-β pathway with a TGFβRI small molecule inhibitor (galunisertib) has been proven to support anti-tumor immunity in a solid tumor study [48]. The treatment with galunisertib showed strong dose-dependence with anti-tumor activities where T cell proliferation mediated by TGF-β was suppressed. Because this effect was CD8+ T cell dependent and experimental results showed a successful establishment of immunological memory, utilizing a secondary immune response to effectively target tumor has become possible. Moreover, the combination of galunisertib with PD-L1 blockade showed a synergic anti-tumor effect [48]. Dissimilar to TGFβ-dependent environment, bone metastasis development starts with colonization of the bone marrow microenvironment. Cells may survive or be dormant depending on the locality. If cells survived a long-lived dormant state, some will re-activate and grow to modify the microenvironment. Eventually, tumor cells become microenvironment independent. Thus, targeting the dependency between colonizing tumor cells and the cells of bone opens new therapeutic opportunities [49].

**Figure 7:**
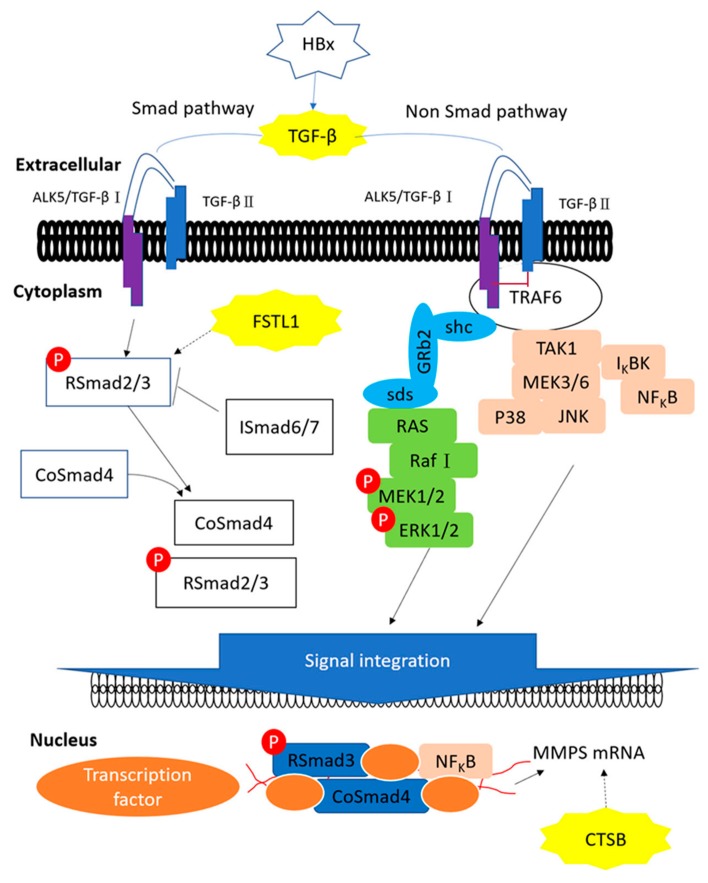
The proposed influences of HBx in TGF-β pathway. HBx, CTSB, TGF-β1 and FSTL1 are heavily involved in the TGF-β signal transmission pathway. HBx induces TGF-β1 to activate Smad and non-Smad pathways where CTSB up-regulates MMP expression and FSTL1 activates RSmad3.

HBx, CTSB, TGF-β1 and FSTL1 are heavily involved in the TGF-β signal transmission pathway (Figure 7). SMAD4 is recognized as an important tumor suppressor, but nuclear SMAD4 levels were significantly increased in HCC tumors [50]. HBx induces TGF-β1 to activate Smad and non-Smad pathways where CTSB up-regulates MMP expression and FSTL1 activates RSmad3. FSTL1 induces liver fibrosis through hepatic stellate cell activation; CTSB plays key roles in extracellular matrix (ECM) degradation and tissue remodeling, which drive hepatic stellate cell proliferation and promote fibrogenic potential [51]. Moreover, Manchanda and co-workers suggested the potential of using CTSB as a diagnostic biomarker for chronic liver diseases [52]. Indeed, these three proteins play important roles in liver fibrosis and our HBx mice did not develop liver tumors.

**Figure 8:**
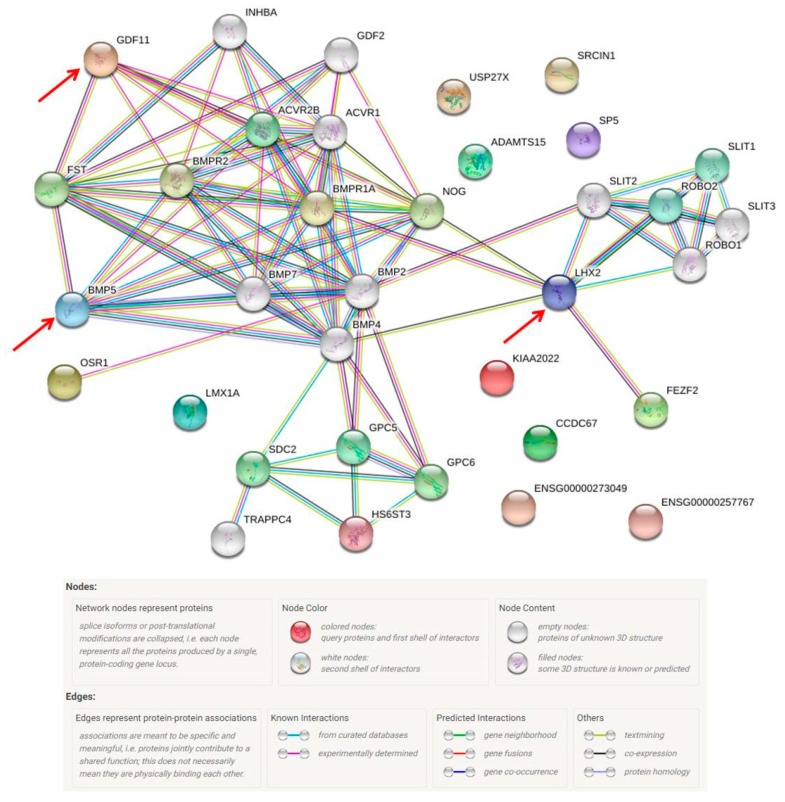
The protein–protein interaction pathways are illustrated. Twenty-nine proteins were identified from the missing protein database to be related with HBX. LHX2, BMP5 and GDF11 are inter-connected with other proteins.

The protein-protein interactions among those 29 proteins are shown in Figure 8. LHX2, BMP5 and GDF11 had complex interactions with other proteins. In the liver, LIM homeobox 2 (LHX2), a transcription factor, is involved in mesoderm development and may function to inhibit the activation of hepatic stellate cells (HSC); such activation is essential to develop hepatic fibrosis and cirrhosis [53]. Growth differentiation factor 11 (GDF11) is also involved in mesoderm and embryonic development. In hepatic tumorigenesis, GDF11 was reported to reduce the viability of liver cancer cells and its mRNA expression was down-regulated in tumor tissues compared with that in the matching normal tissues [54]. After a careful functional assessment of the database, two were found to be related to liver fibrosis and ten were associated with tumorigenesis. Among these 11 proteins, only seven were evident at the transcription level and defined as missing proteins. Bone morphogenetic protein 5 (BMP-5), which was one of the missing proteins (PE2), plays roles in both fibrosis and tumorigenesis.

Bone morphogenetic proteins (BMPs) are members of the TGF-β superfamily known for their roles in dorsal-ventral patterning, organogenesis and cell differentiation [55]. According to their sequence homology and function, the BMP/growth and differentiation factor (GDF) subfamily can be subdivided into seven groups, of which BMP-5, -6, -7 and -8 belong to the same group [56]. It has been shown that BMPs regulate various biological activities, including proliferation, differentiation, migration and cell death. Thus, BMP dysregulation can be pathological. 

Indeed, multifunctional signaling molecules such as BMPs have attracted attention in cancer research, and BMP-5 does have a role in cancer. For example, higher BMP-5 expression is linked to female patients and lung adenocarcinoma (compared to male patients and squamous cell carcinoma), although the overall expression of BMP-5 is inhibited in non-small-cell lung cancer (NSCLC) tissues compared with adjacent normal tissues [57]. As a result, this differential expression pattern might be a potential prognostic biomarker or therapeutic target for patients with NSCLC. In breast cancer, TGF-β1-induced epithelial-to-mesenchymal transition is mediated by Blimp-1-dependent repression of BMP-5. In addition, BMP-5 mRNA levels in breast cancer cell lines and in primary breast tumors were lowered when compared with normal tissues, and correlated with cancer recurrence [58]. In peripheral nerve and neoplastic lesions of nerve sheath tumors, BMP-5 contributed to the maintenance of health, control of proliferation, and neoplastic transformation of the peripheral nervous system. In addition, the BMP-5 mRNA level in malignant schwannoma was relatively lower than that in benign lesions [59]. In adrenocortical carcinoma, BMP-5 was likely to be involved in the modulation of the malignant and functional phenotype of adrenocortical cancer cells. Down-regulation of BMP-5 mRNA expression was found in tissue samples from adrenocortical carcinoma and adrenocortical tumor cell lines when compared with normal adrenal glands [60]. In the digestive system, BMP-5 plays similar roles in both colorectal cancer (CRC) and pancreatic cancer. The loss of BMP-5 expression takes place as an early event in CRC where BMP-5 may dysregulate E-cadherin and then trigger tumor initiation and development [61]. Also, miR32 may target BMP-5 to help CRC development [62]. Low expression levels of BMP-5 were detected in various pancreatic cancer cells compared to normal samples [63]. BMP-5 displayed a biphasic role as being both detrimental and beneficial: BMP-5 treatment inhibited the growth of pancreatic cancer cells but promoted migration and invasion [63].

In the liver, BMPs take part in key functions, including liver specification (BMP-4), control of glucose homeostasis (BMP-4 and -6), liver bud formation (BMP-6), embryonic stem cell differentiation into hepatocyte-like cells (BMP-6), control of iron homeostasis (BMP-6) and enhancement of hepatocyte proliferation during regeneration (BMP-7) [64]. Although the function of BMP-5 in the liver is not clear, it could be similar to that of its groupmates (BMPs 6-8). Our study detected the up-regulation of BMP-5 in liver samples of HBx mice, and these livers had fibrosis but no tumor formation. This oncogenic role is very different from the one previously reported. Jin et al. reported that BMP-5 was down-regulated and acted as a tumor suppressor, and overexpression of BMP-5 was associated with malignancy of the oral epithelium, and especially with metastatic carcinoma cells in lymph nodes [65]. Protein expression of BMP-4 and BMP-7 was upregulated in HBV-related human cirrhosis/HCC samples, and the overexpression of BMP-4 and BMP-7 increased cell viability and enhanced cell migration in HCC cell-lines. [22] Moreover, Chen and coworkers reported that gene amplification and increased gene expression of BMP-5 were significant in HCC samples [66]. This is in agreement with our finding that BMP-5 may contribute to the fibrogenic and tumorigenic processes in the liver.

## 4. Materials and Methods 

### 4.1. Hepatocyte-Specific HBx Transgenic and Wild-Type Mice

The generation of HBx transgenic mice has been described previously [11]. The wild-type C57BL/6 mice were provided by National Laboratory Animal Center (NLAC), NARLabs, Taiwan. All mice were maintained in specific pathogen-free areas of the animal holding facilities and were treated according to protocols approved by the Animal Care and Use Committee of Kaohsiung Medical University. All mice were kept in a 12-hour light–dark cycle room with water and standard mouse pellet chow. The mice sacrificed in this study were 11–13 months old (n = 10 for WT; n = 15 for HBx). All the mice had been fasting for at least 8 hours before sacrifice. 

### 4.2. Serum Alanine Aminotransferase and Aspartate Aminotransferase Tests 

Serum samples without hemolysis were collected for determination of alanine aminotransferase (ALT) and aspartate aminotransferase (AST) activities. Liver transaminases were measured by Hitachi 7080 automatic biochemistry analyzer and were biomarkers of liver injury with some degree of intact liver function. 

### 4.3. Histopathological Examination and Immunohistochemical Staining 

Liver specimens were scored for fibrosis by examining Masson’s trichrome stained slides, and all other parameters, such as degeneration/inflammation, were observed by examining hematoxylin and eosin (H&E) stained slides. The tissues were fixed in 10% formalin, processed, and embedded in paraffin. The tissue slides were sectioned at 3–5 μm in thickness, and stained with H&E or Masson’s trichrome stain using standard procedure. The pathological reports were done by the National Laboratory Animal Center in Taiwan. For immunohistochemical staining analysis, tissue sections were de-paraffinized and subsequent blockage of the endogenous peroxidase activity was achieved by incubation with 2.5% methanolic hydrogen peroxide for 30 min. The tissue sections were stained with antibodies against Hepatitis B Virus X antigen (ab39716), FSTL1 (20182-1-AP), Cathepsin B (PA5-47975) or TGF-β (GTX 108510). The immunohistochemical assays were performed according to the manufacturer’s instructions and counterstained with hematoxylin (Dako). 

### 4.4. Sample Digestion and Preparation 

The mouse livers were homogenized, and then the proteins were extracted with RIPA buffer. Protein samples (100 μL) were transferred into 1.5 mL Eppendorf tubes and incubated at 37 °C for 3 h after mixing with 25 μL of 1 M dithiothreitol (DTT, USB Corporation, 15397). The samples were reduced and alkylated in the dark at room temperature for 30 min after the addition of 25 μL of 1 M iodoacetamide (IAA, Amersham Biosciences, RPN6302V) in 25 mM ammonium bicarbonate. Approximately 10 μL of 0.1 μg/μL modified trypsin digestion buffer (Trypsin Gold, Mass Spectrometry Grade, V5280, Promega, WI, USA) in 25 mM ammonium bicarbonate was added to the protein samples, which were then incubated at 37 °C for at least 12 h in a water bath. Two microliters of formic acid were added to each sample before mass spectrometric analysis for protein identification. 

### 4.5. Immunoblotting

Each tissue lysate sample was electrophoresed through a precast gel (NuPAGE, 4–12% Bis-Tris Gel, 1.5 mm, Invitrogen, Carlsbad, CA). Proteins were transferred from the gel to a polyvinyldifluoride (PVDF) membrane (Millipore, Bedford, CA) and blocked with 5% milk in PBS (adjusted to pH 7.4) containing 0.05% Tween-20. The membranes were then separately incubated overnight with primary antibodies (1 ug/ mL) of anti- FSTL1, anti- Cathepsin B, anti- TGF-β, anti-PREX2 and anti-β-actin. After washing, the membrane was incubated with alkaline peroxidase-conjugated AffiniPure goat anti-rabbit IgG (111-035-003, Immuno Research) for 1 h (1:10,000). Proteins were detected with an enhanced chemiluminescent (ECL) system, and quantitative analysis of Western blotting was carried out using the ImageQuant-TL-7.0 software, version 2010 (Amersham Biosciences). 

### 4.6. Proteomic Analysis 

The protein tryptic digests were fractionated using a flow rate of 400 nL/min with a nano-UPLC system (nanoACQUITY UPLC, Waters, Milford, MA, USA) coupled to an ion trap mass spectrometer (LTQ Orbitrap Discovery Hybrid FTMS, Thermo, San Jose, CA, USA) equipped with an electrospray ionization source. For reverse phase nano-UPLC-ESI-MS/MS analyses, a sample (2 μL) of the desired peptide digest was loaded into the trapping column (Symmetry C18, 5 μm, 180 μm × 20 mm) by an autosampler. Reverse phase separation was performed using a linear acetonitrile gradient from 99% buffer A (100% D.I. water/0.1% formic acid) to 85% buffer B (100% acetonitrile/0.1% formic acid) in 100 min using the micropump at a flow rate of approximately 400 nL/min. Separation was performed on a C18 microcapillary column (BEH C18, 1.7 μm, 75 μm × 100 mm) using the nano separation system. As peptides were eluted from the micro-capillary column, they were electrosprayed into the ESI-MS/MS with the application of a distal 2.1 kV spraying voltage with heated capillary temperature of 200°C. Each scan cycle contained one full-scan mass spectrum (m/z range: 400–2000) and was followed by three data dependent tandem mass spectra. The collision energy of MS/MS analysis was set at 35%. 

### 4.7. Protein Database Search and STRING Database for Protein–Protein Interaction Network Analysis

Mascot software (Version 2.2.1, Matrix Science, London, UK) was used to search the Swiss-Prot protein sequence database. For proteolytic cleavages, only tryptic cleavage was allowed, and the number of maximal internal (missed) cleavage sites was set to 2. There was no modification allowed. Mass tolerances of the precursor peptide ion and fragment ion were set to 10 ppm and 0.5 Da, respectively. In the case of a missing protein identification, we downloaded all gene expression sequences from the human protein atlas web (HPA, www.proteinatlas.org). Search for the presence of any missing protein utilized JAVA programming based on the HPA. The peptide sequences performed by Mascot software were uploaded to our program for missing protein identification and the search algorithm was set to 100% match. When a protein was identified by two or more unique peptides, the protein was considered to be present in the sample. The STRING database accumulates and integrates functional interactions among expressed proteins and consists of direct (physical) and indirect (functional) interactions. The latest version of STRING covers 5090 organisms with 24.6 million proteins. This database is committed to have the maximum protein interactions including primary and predicted interactions, such as annotated pathways, text-mining outcomes, inter-organism transfers or other accessory information [67]. In addition, STRING database was uploaded with differentially expressed genes to the protein-protein interaction (PPI) network. The association evidence of the STRING database can be classified into 7 categories: co-expression, text-mining, experimental data, previously curated pathway and protein-complex knowledge and three predicted categories (neighborhood, fusion, and gene co-occurrence). The STRING version 11 was used for the analysis.

## 5. Conclusions

Proteins significant for the development of liver fibrosis and transition to HCC were suggested and identified by proteomic approaches. The information provided here may shed light on the elucidation of molecular mechanisms involved in HBx-induced HCC. Our approach allows us to identify the critical proteins in hepatocarcinogenesis from an HBx-induced mouse model. These differentially expressed proteins and key missing proteins may serve as useful molecular markers for the early-stage diagnosis or treatment of HCC.

## Figures and Tables

**Table 1:** Summary of proteins corresponding to tumorigenesis in HBx group.

Swiss-Port TrEMBL Accession Number	Protein Name	MW ^a^	Score	Match Queries	p*I* ^b^	Sequence Coverage(%)	Matched Peptide
Q9CQ60	6-phosphogluconolactonase	27237	20	3	5.55	23	R.FALGLSGGSLVSMLAR.D + Oxidation (M); 2 Phospho (ST) R.ILEDKEGTLPAALVQPR.T + Deamidated (NQ) M.AAPAPSLISVFSSPQELGASLAQLVAQR.A + 2 Deamidated (NQ); 3 Phospho (ST)
P10605	Cathepsin B	37256	60	6	5.57	15	K.HEAGDMMGGHAIR.I + Oxidation (M) K.HFGYTSYSVSNSVK.E + 4 Phospho (ST); Phospho (Y) K.NGPVEGAFTVFSDFLTYK.S K.KLCGTVLGGPKLPGR.V + Carboxymethyl (C); Phospho (ST) K.NGPVEGAFTVFSDFLTYK.S + Deamidated (NQ) R.DQGSCGSCWAFGAVEAISDR.T + Carboxymethyl (C)
Q62356	Follistatin-related protein 1	34532	38	1	5.58	2	K.RPVCGSNGK.T + Carboxymethyl (C); Deamidated (NQ)
P06745	Glucose-6-phosphate isomerase	62727	110	3	8.14	5	K.YITKSGAR.V + Phospho (ST); Phospho (Y) K.ILLANFLAQTEALMK.G K.INYTENR.A + Deamidated (NQ)
P19157	Glutathione S-transferase P 1	23594	561	10	7.68	40	R.MLLADQGQSWK.E M.PPYTIVYFPVR.G K.YVTLIYTNYENGK.N + Deamidated (NQ) R.EAAQMDMVNDGVEDLR.G K.FEDGDLTLYQSNAILR.H R.EAAQMDMVNDGVEDLR.G + Oxidation (M) K.AFLSSPEHVNRPINGNGK.Q + 2 Deamidated (NQ) R.EAAQMDMVNDGVEDLR.G + 2 Deamidated (NQ); Oxidation (M) K.FEDGDLTLYQSNAILR.H + Deamidated (NQ) K.AFLSSPEHVNRPINGNGKQ.- + 2 Deamidated (NQ)
Q3UR70	Transforming growth factor-beta receptor-associated protein 1	97241	38	5	6.24	5	K.LKGNAVR.L + Deamidated (NQ) R.DSIHARR.T R.TTQVALGLAK.S R.YPNGGLVHTHCAASR.H + Carboxymethyl (C); Phospho (ST) R.VSTGGKDVEATETQAKLR.R + Deamidated (NQ); 3 Phospho (ST)
P54869	Hydroxymethylglutaryl-CoA synthase	56786	221	7	8.65	21	K.TPMTSQKTFESLVDFCK.T + Carboxymethyl (C); Deamidated (NQ); Oxidation (M); Phospho (ST) K.TPMTSQK.T + Oxidation (M); 3 Phospho (ST) R.LLVPYLIEAVR.L R.QFLRSMSSSSSASAAAK.K + Deamidated (NQ); Oxidation (M); 2 Phospho (ST) R.QFLRSMSSSSSASAAAK.K + Oxidation (M); 4 Phospho (ST) R.SMSSSSSASAAAKK.I + Oxidation (M); 4 Phospho (ST) R.SMSSSSSASAAAK.K + Oxidation (M)

^a^ MW: molecular weight; ^b^ p*I*: isoelectric point.

**Table 2:** Summary of protein function corresponding to tumorigenesis in HBx group.

Name	Subcellular Location	Biological Process	Molecular Function	Protein Function
6-phosphogluconolactonase		Carbohydrate metabolic process		
Cathepsin B	Lysosome	Proteolysis epithelial cell differentiation collagen catabolic process	proteoglycan binding collagen binding	Thiol protease may participate in intracellular degradation and protein turnovers in addition to being involved in tumor invasion and metastasis.
Follistatin-related protein 1	Secreted	Response to starvation	heparin binding	It may regulate some growth factors’ activities on cell proliferation and differentiation.
Glucose-6-phosphate isomerase	Cytoplasm	Positive regulation of immunoglobulin secretion response to immobilization stress	cytokine activity monosaccharide binding ubiquitin protein ligase binding	It is a glycolytic enzyme. It can also function as a tumor-secreted cytokine and an angiogenic factor (AMF) to stimulate endothelial cell motility.
Glutathione S-transferase P 1	Cytoplasm Mitochondrion Nucleus	Negative regulation of fibroblast proliferationcellular response to cell-matrix adhesion	JUN kinase binding kinase regulator activity	
Transforming growth factor-beta receptor-associated protein 1	Cytoplasm	Regulation of transcription	SMAD binding	It is involved in the TGF-beta/activin signaling pathway and linked to complexes of inactive heteromeric TGF-beta and activin receptor through the type II receptor. It can be released upon signaling activation. SMAD4 may play a role in the receptor complex and facilitate its interaction with receptor-regulated Smads, such as SMAD2.
Hydroxymethylglutaryl-CoA synthase	Mitochondrion	Cholesterol biosynthetic process		

**Table 3:** Summary of proteins corresponding to HBV infection.

Swiss-port TrEMBL Accession Number	Protein Name	MW	Score	Match Queries	p*I*	Sequence Coverage (%)	Matched Peptide
Q9CQ60	6-phosphogluconolactonase	27237	20	3	5.55	23	R.FALGLSGGSLVSMLAR.D + Oxidation (M); 2 Phospho (ST) R.ILEDKEGTLPAALVQPR.T + Deamidated (NQ) M.AAPAPSLISVFSSPQELGASLAQLVAQR.A + 2 Deamidated (NQ); 3 Phospho (ST)
P62897	Cytochrome c	11598	71	2	9.61	16	K.GITWGEDTLMEYLENPK.K K.TGQAAGFSYTDANK.N
O08749	Dihydrolipoyl dehydrogenase	54238	63	1	7.99	3	K.IPNIYAIGDVVAGPMLAHK.A
O35382	Exocyst complex component 4	110475	22	5	6.07	9	R.LKEIICEQAAIK.Q + Carboxymethyl (C); Deamidated (NQ) K.VLGVQRPLLQSTIIVEK.T + Deamidated (NQ) -MAAEAAGGKYRSTVSK.S + Oxidation (M); 3 Phospho (ST); Phospho (Y) K.DISAMEEAMSASLQQHK.F + Deamidated (NQ); Oxidation (M); 3 Phospho (ST) K.QCPLREFLTVYIKSIFLNQVLAEINK.E + Phospho (Y)
P06745	Glucose-6-phosphate isomerase	62727	110	3	8.14	5	K.YITKSGAR.V + Phospho (ST); Phospho (Y) K.ILLANFLAQTEALMK.G K.INYTENR.A + Deamidated (NQ)
P11352	Glutathione peroxidase 1	22316	42	4	6.74	18	K.YVRPGGGFEPNFTLFEK.C K.FLVGPDGVPVR.R R.DYTEMNDLQK.R + Oxidation (M); Phospho (ST); Phospho (Y) R.NALPTPSDDPTALMTDPK.Y + Deamidated (NQ); Oxidation (M); Phospho (ST)
P97494	Glutamate--cysteine ligase catalytic subunit	72525	44	4	5.59	6	K.SLFFPDEAINK.H + Deamidated (NQ); Phospho (ST) -.MGLLSQGSPLSWEETQR.H + Deamidated (NQ); Oxidation (M) K.RASGELMTVAR.W + Oxidation (M); Phospho (ST) K.VQLLLNGGDVLETLQEKGER.T + Phospho (ST)
P19157	Glutathione S-transferase P 1	23594	561	10	7.68	40	R.MLLADQGQSWK.E M.PPYTIVYFPVR.G K.YVTLIYTNYENGK.N + Deamidated (NQ) R.EAAQMDMVNDGVEDLR.G K.FEDGDLTLYQSNAILR.H R.EAAQMDMVNDGVEDLR.G + Oxidation (M) K.AFLSSPEHVNRPINGNGK.Q + 2 Deamidated (NQ) R.EAAQMDMVNDGVEDLR.G + 2 Deamidated (NQ); Oxidation (M) K.FEDGDLTLYQSNAILR.H + Deamidated (NQ) K.AFLSSPEHVNRPINGNGKQ.- + 2 Deamidated (NQ)
P62983	Ubiquitin-40S ribosomal protein S27a	17939	52	6	9.68	26	R.TLSDYNIQK.E K.IQDKEGIPPDQQR.L + 2 Deamidated (NQ) K.TITLEVEPSDTIENVK.A K.KSYTTPK.K + Phospho (ST) R.TLSDYNIQK.E R.TLSDYNIQKESTLHLVLR.L + Deamidated (NQ); 2 Phospho (ST); Phospho (Y)
Q3UR70	Transforming growth factor-beta receptor-associated protein 1	97241	38	5	6.24	5	K.LKGNAVR.L + Deamidated (NQ) R.DSIHARR.T R.TTQVALGLAK.S R.YPNGGLVHTHCAASR.H + Carboxymethyl (C); Phospho (ST) R.VSTGGKDVEATETQAKLR.R + Deamidated (NQ); 3 Phospho (ST)
Q9JIQ8	Transmembrane protease serine 2	53491	27	2	8.1	4	R.QSLMFYGSR.H + Deamidated (NQ); Oxidation (M); Phospho (ST) K.SSGALCTSKSK.K + Carboxymethyl (C); 2 Phospho (ST)

**Table 4:** Summary of protein function corresponding to HBV infection.

Name	Subcellular Location	Biological Process	Molecular Function	Function
6-phosphogluconolactonase		carbohydrate metabolic process		
Cytochrome c	Mitochondrion	apoptotic process hydrogen peroxide metabolic process mitochondrial electron transport	electron transporter heme binding	This protein has a role in apoptosis. If suppresses the anti-apoptotic members or activates the pro-apoptotic members of the Bcl-2 family, this will lead to altered mitochondrial membrane permeability and result in releasing cytochrome c into the cytosol. When cytochrome c binds to Apaf-1, the activation of caspase-9 will be triggered and then accelerating apoptosis by activating other caspases
Dihydrolipoyl dehydrogenase	Mitochondrion matrix Nucleus	mitochondrial electron transport cell redox homeostasis lipoate metabolic process regulation of membrane potential	electron transfer activity	
Exocyst complex component 4		chemical synaptic transmission protein targeting to membrane	Ral GTPase binding protein N-terminus binding	It is part of the exocyst complex involved in the docking of exocytic vesicles and may have fusion sites on the plasma membrane.
Glucose-6-phosphate isomerase	Cytoplasm	positive regulation of immunoglobulin secretion response to immobilization stress	cytokine activity monosaccharide binding ubiquitin protein ligase binding	It is a glycolytic enzyme. It can also function as a tumor-secreted cytokine and an angiogenic factor (AMF) to stimulate endothelial cell motility.
Glutathione peroxidase 1	Cytoplasm	response to hydroperoxide regulation of neuron apoptotic process protein oxidation triglyceride metabolic process	SH3 domain binding	The oxidative breakdown to the hemoglobin in erythrocytes can be protected by this protein.
Glutamate--cysteine ligase catalytic subunit	Cytosol	cysteine metabolic process negative regulation of extrinsic apoptotic signaling pathway negative regulation of hepatic stellate cell activation	ADP binding ATP binding coenzyme binding	
Glutathione S-transferase P 1	Cytoplasm Mitochondrion Nucleus	negative regulation of fibroblast proliferationcellular response to cell-matrix adhesion	JUN kinase binding kinase regulator activity	
Ubiquitin-40S ribosomal protein S27a	Cytoplasm Nucleus	translation	metal ion binding structural constituent of ribosome	
Transforming growth factor-beta receptor-associated protein 1	Cytoplasm	regulation of transcription	SMAD binding	It is involved in the TGF-beta/activin signaling pathway and linked to complexes of inactive heteromeric TGF-beta and activin receptor through the type II receptor. It can be released upon signaling activation. SMAD4 may play a role in the receptor complex and facilitate its interaction with receptor-regulated Smads, such as SMAD2.
Transmembrane protease serine 2	Cell membrane	protein autoprocessing	scavenger receptor activity	It proteolytically cleaves and activates the glycoproteins of viral spikes and then facilitates virus-cell membrane fusions. Because such spike proteins are synthesized and maintained in the intermediate folding states, this proteolysis permits the refolding and energy release. As a result, this creates stable virus-cell linkages and membrane coalescence.

**Table 5:** Summary of proteins corresponding to inflammation.

Swiss-port TrEMBL Accession Number	Protein Name	MW	Score	Match Queries	p*I*	Sequence Coverage(%)	Matched Peptide
P11352	Glutathione peroxidase 1	22316	42	4	6.74	18	K.YVRPGGGFEPNFTLFEK.C K.FLVGPDGVPVR.R R.DYTEMNDLQK.R + Oxidation (M); Phospho (ST); Phospho (Y) R.NALPTPSDDPTALMTDPK.Y + Deamidated (NQ); Oxidation (M); Phospho (ST)
P16627	Heat shock 70 kDa protein 1-like	70593	98	15	5.91	12	K.VMVSYKGEK.K + Phospho (Y) K.VMVSYKGEK.K + Oxidation (M); Phospho (Y) K.VEIIANDQGNR.T R.KELENMCNPIITKLYQSGCTGPTCTPGYTPGR.A + 2 Carboxymethyl (C); 2 Deamidated (NQ); Oxidation (M); 4 Phospho (ST); Phospho (Y) K.AFYPEEISSMVLTKMK.E + Phospho (ST) R.KELENMCNPIITKLYQSGCTGPTCTPGYTPGR.A + 2 Deamidated (NQ); Oxidation (M); 5 Phospho (ST); 2 Phospho (Y) K.MDKAKIHDIVLVGGSTR.I + Oxidation (M); Phospho (ST) K.LWPFQVINEAGKPKVMVSYKGEK.K + 2 Deamidated (NQ); Oxidation (M); Phospho (Y) K.VMVSYKGEK.K + Phospho (ST) K.ITITNDKGRLSK.E + Deamidated (NQ); 2 Phospho (ST) K.NQVAMNPQNTVFDAK.R + Phospho (ST) K.NALESYAFNMK.S + Deamidated (NQ); Oxidation (M); Phospho (ST); Phospho (Y) R.TTPSYVAFTDTER.L K.AFYPEEISSMVLTK.M + Oxidation (M) K.SINPDEAVAYGAAVQAAILMGDK.S + Phospho (ST)
P11499	Heat shock protein HSP 90-beta	83229	175	21	4.97	18	R.YESLTDPSK.L K.YIDQEELNK.T K.ADLINNLGTIAK.S R.ELISNASDALDK.I K.EDQTEYLEER.R K.EISDDEAEEEK.G + Phospho (ST) K.IEDVGSDEEDDSGK.D R.GVVDSEDLPLNISR.E K.NIVKKCLELFSELAEDK.E + Carboxymethyl (C); Phospho (ST) K.VTISNRLVSSPCCIVTSTYGWTANMER.I + 2 Carboxymethyl (C); 2 Deamidated (NQ); Oxidation (M); 3 Phospho (ST); Phospho (Y) R.EMLQQSK.I + Phospho (ST) K.LMKEILDKK.V R.DNSTMGYMMAK.K + Oxidation (M) K.AQALRDNSTMGYMMAK.K + 2 Deamidated (NQ); 2 Oxidation (M); Phospho (ST) K.VTISNRLVSSPCCIVTSTYGWTANMER.I + Oxidation (M); Phospho (ST) K.FENLCKLMK.E R.IMKAQALRDNSTMGYMMAK. K + Deamidated (NQ); 3 Oxidation (M); Phospho (ST) R.TLTLVDTGIGMTKADLINNLGTIAK.S + Oxidation (M); 4 Phospho (ST) R.TLTLVDTGIGMTK.A + Oxidation (M); 2 Phospho (ST) K.IEDVGSDEEDDSGK.D + Phospho (ST) K.AQALRDNSTMGYMMAKK.H + Deamidated (NQ); Oxidation (M); 2 Phospho (ST); Phospho (Y)
P63017	Heat shock cognate 71 kDa protein	70827	101	20	5.37	16	K.VEIIANDQGNR.T K.STAGDTHLGGEDFDNR.M K.STGKENKITITNDK.G + Deamidated (NQ); 3 Phospho (ST) K.NSLESYAFNMKATVEDEK.L + Oxidation (M); Phospho (ST) K.NSLESYAFNMKATVEDEK.L + Deamidated (NQ); Oxidation (M); Phospho (Y) K.SINPDEAVAYGAAVQAAILSGDK.S K.TVTNAVVTVPAYFNDSQRQATK.D + 4 Deamidated (NQ); Phospho (ST) M.SKGPAVGIDLGTTYSCVGVFQHGK.V + 2 Phospho (ST); Phospho (Y) M.SKGPAVGIDLGTTYSCVGVFQHGK.V + Deamidated (NQ); 2 Phospho (ST); Phospho (Y) K.LQGKINDEDKQK.I + 2 Deamidated (NQ) K.STGKENKITITNDK.G + 3 Phospho (ST) K.ITITNDKGRLSK.E + Deamidated (NQ); 2 Phospho (ST) K.TVTNAVVTVPAYFNDSQR.Q + 3 Deamidated (NQ); 4 Phospho (ST) K.EIAEAYLGK.T + Phospho (Y) K.QTQTFTTYSDNQPGVLIQVYEGER.A + 3 Deamidated (NQ); Phospho (ST) K.EIAEAYLGK.T K.LLQDFFNGK.E + 2 Deamidated (NQ) R.AMTKDNNLLGK.F + 2 Deamidated (NQ); Phospho (ST) M.SKGPAVGIDLGTTYSCVGVFQHGK.V + Deamidated (NQ); 3 Phospho (ST); Phospho (Y) R.TTPSYVAFTDTER.L
Q3UR70	Transforming growth factor-beta receptor-associated protein 1	97241	38	5	6.24	5	K.LKGNAVR.L + Deamidated (NQ) R.DSIHARR.T R.TTQVALGLAK.S R.YPNGGLVHTHCAASR.H + Carboxymethyl (C); Phospho (ST) R.VSTGGKDVEATETQAKLR.R + Deamidated (NQ); 3 Phospho (ST)
Q9JIQ8	Transmembrane protease serine 2	53491	27	2	8.1	4	R.QSLMFYGSR.H + Deamidated (NQ); Oxidation (M); Phospho (ST) K.SSGALCTSKSK.K + Carboxymethyl (C); 2 Phospho (ST)
P54869	Hydroxymethylglutaryl-CoA synthase	56786	221	7	8.65	21	K.TPMTSQKTFESLVDFCK.T + Carboxymethyl (C); Deamidated (NQ); Oxidation (M); Phospho (ST) K.TPMTSQK.T + Oxidation (M); 3 Phospho (ST) R.LLVPYLIEAVR.L R.QFLRSMSSSSSASAAAK.K + Deamidated (NQ); Oxidation (M); 2 Phospho (ST) R.QFLRSMSSSSSASAAAK.K + Oxidation (M); 4 Phospho (ST) R.SMSSSSSASAAAKK.I + Oxidation (M); 4 Phospho (ST) R.SMSSSSSASAAAK.K + Oxidation (M)

**Table 6:** Summary of protein function corresponding to inflammation.

Name	Subcellular Location	Biological Process	Molecular Function	Function
Glutathione peroxidase 1	Cytoplasm	Response to hydroperoxide regulation of neuron apoptotic process protein oxidation triglyceride metabolic process	SH3 domain binding	Protects the hemoglobin in erythrocytes from oxidative breakdown.
Heat shock 70 kDa protein 1-like	Mitochondrion Nucleus	Cell differentiation protein refolding	ATP binding unfolded protein binding	It may play a pivotal role in the protein quality control system, ensuring the correct folding of proteins, the re-folding of misfolded proteins and controlling the targeting of proteins for subsequent degradation.
Heat shock protein HSP 90-beta	Cell membrane Cytoplasm Membrane Nucleus	Positive regulation of telomerase activity positive regulation of protein binding	ATP binding GTP binding protein kinase binding	Molecular chaperone that promotes the maturation, structural maintenance and proper regulation of specific target proteins involved for instance in cell cycle control and signal transduction.
Heat shock cognate 71 kDa protein	Cell membrane Cytoplasm Nucleus	ATP metabolic process regulation of cell cycle protein folding	ATP binding enzyme binding	Molecular chaperone implicated in a wide variety of cellular processes, including protection of the proteome from stress, folding and transport of newly synthesized polypeptides, activation of proteolysis of misfolded proteins and the formation and dissociation of protein complexes. Plays a pivotal role in the protein quality control system, ensuring the correct folding of proteins, the re-folding of misfolded proteins and controlling the targeting of proteins for subsequent degradation. This is achieved through cycles of ATP binding, ATP hydrolysis and ADP release, mediated by co-chaperones.
Transforming growth factor-beta receptor-associated protein 1	Cytoplasm	Regulation of transcription	SMAD binding	It may plays a role in the TGF-beta/activin signaling pathway. It associates with inactive heteromeric TGF-beta and activin receptor complexes, mainly through the type II receptor, and is released upon activation of signaling. May recruit SMAD4 to the vicinity of the receptor complex and facilitate its interaction with receptor-regulated Smads, such as SMAD2.
Transmembrane protease serine 2	Cell membrane	Protein autoprocessing	scavenger receptor activity	Serine protease that proteolytically cleaves and activates the viral spike glycoproteins which facilitate virus-cell membrane fusions. The spike proteins are synthesized and maintained in precursor intermediate folding states and proteolysis permits the refolding and energy release required to create stable virus-cell linkages and membrane coalescence.
Hydroxymethylglutaryl-CoA synthase	Mitochondrion	Cholesterol biosynthetic process	hydroxymrthylglutaryl-CoA synthase activity	This enzyme condenses acetyl-CoA with acetoacetyl-CoA to form HMG-CoA, which is the substrate for HMG-CoA reductase.

**Table 7:** Summary of proteins identified by the human protein atlas (HPA) database.

Serial No.	Chromosome No.	Gene Name	Gene ID	Chromosome Position	Transcript ID	Protein Class	Protein Evidence	Matched Sequence
1	7	AC068533.7	ENSG00000249319	66087761–66152277	ENST00000450043	Predicted intracellular proteins	Evidence at transcript level	RCAGLLMTLKGLPSTYNKD
								RQAHEASGKA
								KNPDSLELIR
								RCAGLLMTLK
								RDFVAEFLFWASLCMTHLSR
2	17	BAHCC1-201	ENSG00000266074	1399740–81466332	ENST00000307745	Predicted intracellular proteins	Evidence at transcript level	KHLTSCLLNTKV
								SPTPPPR
								RSPARRGPGRPRK
								KLDHEGVTSPKN
								KHLTSCLLNTK
								KWSGNPTQR
								KWSGNPTQRR
								RRGSPLLSWSAVAQTK
								RSPARRGPGRPR
								KEALSFSKAKELSR
								KERQGLLGACR
								KLDHEGVTSPK
3	4	CCSER1-003	ENSG00000184305	90127535–90923530	ENST00000505073	Predicted intracellular proteins	Evidence at transcript level	SSSEGTAGSSR
								ITRSLTEDFEREK
								TNSPR
								LRSSSEGTAGSS
								SSSEGTAGSS
4	13	KLHL1	ENSG00000150361	69700594–70108493	ENST00000377844	Predicted intracellular proteins	Evidence at transcript level	KSTVGTLYAVGGMDNNKGATTIEK
								STVGTLYAVGGMDNNK
								LQQGAPGQGTQQPA
								NDLECQ
5	3	KLHL24-001	ENSG00000114796	183635568–183684477	ENST00000242810	Predicted intracellular proteins	Evidence at transcript level	DVWIYNSQLNIWIR
								INSRDVWIYNSQLNIWIR
								QENCGMSVCNG
								VQSYDPETNSWLL
6	12	RP11-162P23.2	ENSG00000257767	111753890–111791418	ENST00000546840	Predicted intracellular proteins	Evidence at transcript level	RTYLAALETLDNGKPYVISYLVDLDMVLKC
								KTIPIDGDFFSYTRH
								KLGPALATGNVVVMKV
								KVAEQTPLTALYVANLIKE
								RAAFQLGSPWRR
								KLGPALATGNVVVMK
								KTIPIDGDFFSYTR
								KVAEQTPLTALYVANLIK
								RAAFQLGSPWR
								RLADLIER
								KTIPIDGDFFSYTR
7	17	SRCIN1-001	ENSG00000277363	38530016–38605930	ENST00000617146	Predicted intracellular proteins	Evidence at protein level	MEAMEKQIASLTGLVQSALLR
								TGEVVVTSK
								SRLSYAGGRPPSYAGSPVHHAAER
								RCRGVTDTLAQI
								REMVYAS
8	11	ADAMTS15	ENSG00000166106	130448974–130476641	ENST00000299164	Enzymes, predicted secreted proteins	Evidence at transcript level	SFREEQCEAFNGYNHSTNR
								EEQCEAFNGYNHSTNR
9	12	ANO4-002	ENSG00000151572	100717526–101128641	ENST00000392977	Predicted intracellular proteins, predicted membrane proteins	Evidence at transcript level	DLRDRMR
								LINRWR
10	6	BMP5	ENSG00000112175	55753645–55875564	ENST00000370830	Predicted intracellular proteins	Evidence at transcript level	LNAISVLYFDDSSNVILK
								TPLTTQSPPLASLHDTNFLNDADMVMSFVNLVER
11	11	CCDC67-002	ENSG00000165325	93329990–93415200	ENST00000530862	Predicted intracellular proteins, predicted membrane proteins	Evidence at protein level	SELQSRDDLLR
								MYQRQCQAMEAGLSEVK
12	3	FEZF2-004	ENSG00000153266	62369681–62374324	ENST00000486811	Predicted intracellular proteins, transcription factors	Evidence at transcript level	IIHTQEKPHKCNQCGK
								KLLNLCSPLPCMIPLQPLGYEVPSK
13	15	GABRG3-001	ENSG00000182256	26971282–27541991	ENST00000615808	FDA approved drug targets Predicted intracellular proteins, predicted membrane proteins Predicted secreted proteins	Evidence at protein level	IWNDGK
								KNSVEAADQK
14	12	GDF11-001	ENSG00000135414	55743280–55757278	ENST00000257868	Predicted secreted proteins	Evidence at protein level	RNLGLDCDEHSSESR
								SLKIELHSR
15	13	HS6ST3	ENSG00000185352	96090839–96839562	ENST00000376705	Predicted intracellular proteins, predicted secreted proteins	Evidence at transcript level	HLVKNIR
								RHLVKNIRL
16	23X	KIAA2022-001	ENSG00000050030	74732849–74925447	ENST00000055682	Disease related genes, predicted intracellular proteins	Evidence at transcript level	DCSRYMARDTNSGSSSSQQNYGLR
								YMARDTNSGSSSSQQNYGLR
								APFAIMEPAGMSALNGDCLMQPSRTCLGCFMESK
								HKSSSK
17	9	LHX2-004	ENSG00000106689	124001670–124015361	ENST00000560961	Predicted intracellular proteins, transcription factors	Evidence at transcript level	CARCHLGISASEMVMR
								CHLGISASEMVMRAR
18	1	LMX1A-001	ENSG00000162761	165201867–165356715	ENST00000342310	Predicted intracellular proteins, transcription factors	Evidence at protein level	EPLETTCFYRDKK
								KVRETLAAETGLSVR
19	19	LRFN3-001	ENSG00000126243	35935550–35945767	ENST00000588831	Predicted intracellular proteins, transcription factors	Evidence at transcript level	LDMTSNR
								LARLDMTSNR
								LDMTSNRLTTIPPDPLFSRLPLLARPR
20	6	MSH5-SAPCD1-001	ENSG00000255152	31740020–31764851	ENST00000493662	Plasma proteins, predicted intracellular proteins, predicted secreted proteins	Evidence at protein level	DQETLLMYQLQCQVLAR
								IHSCESISLGLSTFMIDLNQVAK
21	2	OSR1	ENSG00000143867	19351485–19358653	ENST00000272223	Enzymes, predicted intracellular proteins	Evidence at protein level	EFVCKFCGR
								TSKIKC
								GFCQSRTLAVHKTLHSQVK
22	17	RASL10B	ENSG00000270885	35731649–35743521	ENST00000603017	Predicted intracellular proteins	Evidence at protein level	KTWKCGYVECSAK
								WNVSHLVRKTWK
23	2	RNF103-CHMP3-002	ENSG00000249884	86505668–86721122	ENST00000604011	Predicted intracellular proteins	Evidence at transcript level	NQLAVLRVAGSLQK
								AHMNSVLMGMKNQLAVLR
24	12	RP11-834C11.12	ENSG00000273049	53985845–54034888	ENST00000513209	Predicted intracellular proteins	Evidence at transcript level	TSYTRYQTLELEK
								SDSQTPSPNEIK
25	2	SP5	ENSG00000204335	170715351–170718078	ENST00000375281	Predicted intracellular proteins, transcription factors	Evidence at transcript level	NDSLQAFLQDRTPSASPDLGK
								FACPECGK
26	2	SP9	ENSG00000217236	174334946–174338492	ENST00000394967	Predicted intracellular proteins, transcription factors	Evidence at transcript level	THNGGGGGKKGSDSDTDASNLETPR
								LGPAGASLRRK
								RYSGRATCDCPNCQEAER
								MATSILGEEPRFGTTPLAMLAATCNK
27	1	TSSK3-001	ENSG00000162526	32362197–32364312	ENST00000373534	Enzymes, predicted intracellular proteins	Evidence at protein level	MEDFLLSNGYQLGKTIGEGTYSK
								TLDHKNIIQVYEMLESADGK
28	23X	USP27X	ENSG00000273820	49879948–49882565	ENST00000621775	Enzymes, predicted intracellular proteins	Evidence at transcript level	CGSCQSYQESTK
								ITTYISFPLELDMTPFMASSK
29	23Y	USP9Y	ENSG00000279327	12709448–12859416	ENST00000625141	Disease related genes, enzymes, potential drug targets, predicted intracellular proteins	Evidence at protein level	FFRDGLTISFTK
								ASWTNASKK
								LYSVVSQLIR
								MDDDEEMK

**Table 8:** Summary of protein function corresponding to tumorigenesis.

UniProtKBEntry	Gene Name	UniProt Protein Name	Subcellular Location	Biological Process	Molecular Function	Protein Function
Q7M6Y5	CCDC67-002	Deuterosome assembly protein 1	Cytoplasm	Cilium biogenesis/degradation	Identical protein binding	Key structural component of the deuterosome, a structure that promotes de novo centriole amplification in multiciliated cells. Deuterosome-mediated centriole amplification occurs in terminally differentiated multiciliated cells and can generate more than 100 centrioles. Probably sufficient for the specification and formation of the deuterosome inner core. Interacts with CEP152 and recruits PLK4 to activate centriole biogenesis.
Q9Z1W4	GDF11-001	Growth/differentiation factor 11	Secreted	Animal organ morphogenesis, cell development, negative regulation of cell differentiation	Cytokine, Growth factor	Secreted signal that acts globally to specify positional identity along the anterior/posterior axis during development (PubMed:10391213). May play critical roles in patterning both mesodermal and neural tissues and in establishing the skeletal pattern. Signals through activin receptors type-2, ACVR2A and ACVR2B, and activin receptors type-1, ACVR1B, ACVR1C and TGFBR1 leading to the phosphorylation of SMAD2 and SMAD3 (PubMed:16845371, PubMed:12414726).
Q9Z0S2	LHX2-004	LIM/homeobox protein Lhx2	Nucleus	Activator, DNA-binding	Transcription, Transcription regulation	Acts as a transcriptional activator. Stimulates the promoter of the alpha-glycoprotein gene. Transcriptional regulatory protein involved in the control of cell differentiation in developing lymphoid and neural cell types.
P49003	BMP5	Bone morphogenetic protein 5	Secreted	Chondrogenesis, Differentiation, Osteogenesis	Cytokine, Developmental protein, Growth factor	Induces cartilage and bone formation.
Q9ESP5	FEZF2-004	Fez family zinc finger protein 2	Nucleus	Differentiation, Neurogenesis, Transcription, Transcription regulation	Developmental protein, DNA-binding, Repressor	Transcription repressor. Required for the specification of corticospinal motor neurons and other subcerebral projection neurons. May play a role in layer and neuronal subtype-specific patterning of subcortical projections and axonal fasciculation. Controls the development of dendritic arborization and spines of large layer V pyramidal neurons. Plays a role in rostro-caudal patterning of the diencephalon and in prethalamic formation.
Q9QYK4	HS6ST3	Heparan-sulfate 6-O-sulfotransferase 3	Membrane Single-pass type II membrane protein	Blastocyst hatching, glycosaminoglycan biosynthetic process	Transferase	6-O-sulfation enzyme which catalyzes the transfer of sulfate from 3’-phosphoadenosine 5’-phosphosulfate (PAPS) to position 6 of the N-sulfoglucosamine residue (GlcNS) of heparan sulfate.
Q5DTT1	KIAA2022-001	Neurite extension and migration factor	Nucleus Cytoplasm	Neurogenesis, Transcription, Transcription regulation	Developmental protein	Involved in neurite outgrowth by regulating cell-cell adhesion via the N-cadherin signaling pathway. May act by regulating expression of protein-coding genes, such as N-cadherins and integrin beta-1 (ITGB1).
Q9JHX2	SP5	Transcription factor Sp5	Nucleus	Transcription, Transcription regulation	Activator, DNA-binding	Binds to GC boxes promoters elements. Probable transcriptional activator that has a role in the coordination of changes in transcription required to generate pattern in the developing embryo.
P59384	ADAMTS15	A disintegrin and metalloproteinase with thrombospondin motifs 15	extracellular marix	Metalloendopeptidase activity	Hydrolase, Metalloprotease, Protease	This gene encodes a member of the ADAMTS (a disintegrin and metalloproteinase with thrombospondin motifs) protein family. ADAMTS family members share several distinct protein modules, including a propeptide region, a metalloproteinase domain, a disintegrin-like domain, and a thrombospondin type 1 (TS) motif. Individual members of this family differ in the number of C-terminal TS motifs, and some have unique C-terminal domains. The encoded preproprotein is proteolytically processed to generate the mature enzyme, which may play a role in versican processing during skeletal muscle development. This gene may function as a tumor suppressor in colorectal and breast cancers.
Q9WVG7	OSR1	Protein odd-skipped-related 1	Nucleus	Transcription, Transcription regulation	Developmental protein, DNA-binding	Transcription factor that plays a role in the regulation of embryonic heart and urogenital development.
Q8CEG8	USP27X	Ubiquitin carboxyl-terminal hydrolase 27	Cytosol Nucleus	Ubl conjugation pathway	Hydrolase, Protease, Thiol protease	Deubiquitinase that can reduce the levels of BCL2L11/BIM ubiquitination and stabilize BCL2L11 in response to the RAF-MAPK-degradation signal. By acting on BCL2L11 levels, may counteract the anti-apoptotic effects of MAPK activity.
Q9JKU8	LMX1A-001	LIM homeobox transcription factor 1-alpha	Nucleus	Activator, Developmental protein, DNA-binding	Transcription, Transcription regulation	Acts as a transcriptional activator by binding to an A/T-rich sequence, the FLAT element, in the insulin gene promoter. Required for development of the roof plate and, in turn, for specification of dorsal cell fates in the CNS and developing vertebrae.
Q9Z0S2	LHX2-004	LIM/homeobox protein Lhx2	Nucleus	Transcription, Transcription regulation	Activator, DNA-binding	Acts as a transcriptional activator. Stimulates the promoter of the alpha-glycoprotein gene. Transcriptional regulatory protein involved in the control of cell differentiation in developing lymphoid and neural cell types.

## Supplementary Materials

The following are available online at https://www.mdpi.com/2072-6694/12/2/409/s1, Figure S1: Serum alanine aminotransferase (ALT) and aspartate aminotransferase (AST) level measurements showed that the averages of AST and ALT in the HBx transgenic mice were significantly higher than that in the wild-type mice. Figure S2. Immunoblotting of mouse liver samples. The PREX2 protein expression levels from HBx transgenic mice are higher than that from WT mice.
